# A Smartphone Ecological Momentary Assessment/Intervention “App” for Collecting Real-Time Data and Promoting Self-Awareness

**DOI:** 10.1371/journal.pone.0071325

**Published:** 2013-08-14

**Authors:** Jason D. Runyan, Timothy A. Steenbergh, Charles Bainbridge, Douglas A. Daugherty, Lorne Oke, Brian N. Fry

**Affiliations:** 1 Behavioral Science Division, Indiana Wesleyan University, Marion, Indiana, United States of America; 2 The Center for Learning and Innovation, Indiana Wesleyan University, Marion, Indiana, United States of America; University of California, San Francisco, United States of America

## Abstract

We have designed a flexible ecological momentary assessment/intervention smartphone (EMA/EMI) “app”. We examine the utility of this app for collecting real-time data, and assessing intra-subject variability, by using it to assess how freshman undergraduates spend their time. We also explore whether its use can promote greater self-awareness. Participants were randomly divided into an experimental group, who used the app, and a control group, who did not. We used the app to collect both randomized in-the-moment data as well as end-of-day data to assess time use. Using a posttest survey we asked participants questions about how they spent time throughout the school semester. We also asked the experimental group about their experience with the app. Among other findings, 80.49% participants indicated that they became more aware of how they spent their time using the app. Corroborating this report, among the experimental group, end-of-semester self-assessment of time spent wasted, and time spent using electronics recreationally, predicted semester GPA at a strength comparable to high school GPA and ACT score (two of the best single predictors for first semester college GPA), but had no correlation among controls. We discuss the advantages and limitations of using apps, such as ours, for EMA and/or EMI.

## Introduction

Much of what we have learned about people through quantitative psychological studies is not the result of studying psychological phenomena as it occurs within everyday life, but, rather, has involved laboratory experiments and questionnaires. Since the beginning of psychological science it has, however, been well known that these traditional methods have limitations, e.g., [1], [2]. First, there are questions concerning *ecological validity*, or the extent to which what is learned in a laboratory setting, or in the setting within which a questionnaire is administered, can be generalized to everyday life [3], [4]. Second, *retrospective or generalized responses* are, to a significant degree, inaccurate as a result of limitations in autobiographical memory whereby respondents must rely on estimation, extrapolation and inference strategies that are inherently unreliable [5]. Additionally, there are dynamic processes (e.g., changes in level of pain or anxiety) that cannot be accurately assessed within everyday life, or correlated with other concurrent experiences, using retrospective or generalized responses. Third, traditional methods often do not capture *intra-subject variability* [6]. As a result of this limitation there has been an inability to measure construct variability (and test the significance of this variability) as well as a tendency to assume that constructs are stable.

In an effort to address the limitations associated with traditional quantitative methods, there has been a growing interest in developing innovative approaches to assessing psychological constructs in real-time, or within daily life. Such methods go by a variety of names (e.g., experience sampling, ambulatory assessment), but, for the purpose of this study, we will refer to them as *ecological momentary assessment* (EMA). As mobile electronic technologies advance, potential approaches to EMA expand. Within the last four years alone, mobile electronic devices have been used to study substance abuse [7], self-injurious thoughts and behavior [8], levels of stress amongst physicians [9], psychopathology [10], affective and cognitive responses amongst stutterers [11], levels of pain amongst chiropractic patients [12], [13], emotional stress and pain amongst patients of breast cancer and their partners [14], diet adherence amongst adolescent diabetics [15], levels of physical activity [16], emotional states [17], and fatigue amongst osteoarthritis patients [18].

In addition to allowing new approaches to EMA, advances in handheld technology allow new ways for clinicians to intervene in the lives of their clients in real-time; that is, it allows for *ecological momentary intervention* (EMI). There is a growing body of research indicating that changes in self-awareness, and becoming more mindful of oneself and one’s responses, promotes positive behavioral and cognitive change, cf. [19]. Within the context of these findings, it has been theorized that being asked questions about one’s behavior, thought patterns or states in close contextual and temporal proximity to their occurrence draws one’s attention toward their occurrence, thereby promoting self-awareness; and, in turn, positive change with respect to focal behaviors, thought patterns or states [7]. It has, thus, been theorized that EMA/EMI may be particularly effective in prompting behavioral and cognitive change in this way. Indeed, recent studies indicate that the use of mobile electronic devices helps with smoking cessation [20], [21], [22], facilitates emotional regulation [23], and facilitate prevention behavior amongst HIV-infected individuals [24], [25].

All of the EMA/EMI studies mentioned above–and all that we are aware of to date save one using a smartphone app designed specifically for Borderline Personality Disorders [26]–have relied on cell phone calls, text messages, or handheld computers, each of which come with inherent limitations that have constrained the use of EMA/EMI. The use of cell phone calls and text messages requires the use of cell phone plans, and requires researchers to place calls and texts either manually, or with an automated system. Further, the coding of this data can be unwieldy and, as a result, be a delimiting factor. The introduction of handheld computers, or PDAs, into daily life is intrusive, and requires participants to become familiar with and carry a device they otherwise would not carry. Additionally, this approach is costly, requires programming expertise, and obliges researchers to personally give, or send, a device to each participant and show them with how to use it.

Here, we introduce a dynamic EMA/EMI platform that capitalizes on iPhone, iTouch and iPad application (“app”) technology. We do so by reporting an experimental study we implemented to assess how first semester undergraduates spent their time. The app platform we introduce in this paper addresses the above mentioned problems associated with current EMA/EMI approaches, and is designed to be flexible so that it can be programmed and used to (i) generate data pertaining to enumerable research questions, and (ii) potentially modify enumerable thought patterns and/or behaviors. One of the main advantages of using an EMA/EMI app is that no data plan or internet connection are required during data collection–it can collect data “off-line”. It also allows researchers to collect randomized data within specified hours. And, though there are a number of apps that have been designed to collect and provide summary data (e.g., “Way of Life”, “The Habit Factor”, “i Run, You Run”), to our knowledge our app is the first designed specifically for flexible EMA/EMI use; and the first to be used for that expressed purpose. For research purposes, our EMA/EMI app has the advantage of allowing participants to use their own devices, cf. [27], [28]; as well as allowing researchers, or clinicians, to specify the exact question types, when they would like participants to be queried, collect data “off-line”, and to have access to the raw data, which includes data time-stamps. This last feature allows researchers to know exactly when a response has been recorded, which has been an issue for certain approaches to EMA, cf. [29].

With the widespread use of smartphones, this app platform makes EMA/EMI more practical for multiple purposes and in multiple contexts. As recently recognized by Miller [30], app platforms–like the one we introduce here–have the potential of expanding the use of a more acute and powerful approach to psychological science. And, as recognized by Cohn et al. [31], apps also have the potential to expand the ability to directly intervene within people’s lives in order to promote positive change. Recent data on cell phone ownership underscores the potential for smartphone apps designed to expand and apply psychological knowledge. 46% of U.S. adults own a smartphone and rates of ownership are even higher and growing faster among young adults [32]. So the large installed base of smartphone owners, coupled with ever expanding phone capabilities, makes apps like the EMA/EMI platform we describe here attractive tools for researchers.

The primary aim of the present study is to demonstrate the feasibility of using the EMA/EMI app platform we have designed for collecting real-time data, and assessing intra-subject variability. Our secondary aim is to explore whether the app can be used to promote change in self-awareness; and whether it can do so even amongst individuals not expressly seeking change. Specifically, we test whether using the app to assess how participants spend their time promotes self-awareness of time spent. Finding out that it does would indicate that this platform has the potential of being used for multiple clinical, and practical, applications as it provides a means by which a changeable set of cues can be sent to participants–and real-time data collected–for the purpose of increasing self-awareness, and thereby fostering positive change for those interested in changing.

## Methods

### Ethics Statement

This research study was approved by the Indiana Wesleyan University Institutional Review Board (IRB). All participants were made aware of what participation would entail and provided written consent. Academic information was obtained only with written consent, and personal information was kept confidential. We also received consent via email or text from the parents of two participants who were 17 years old, and, thus, legal minors at the beginning of the study. Receiving consent in this manner was, given the parameters of the study, the only feasible way to not preclude any students who were less than 18 when the study began. These parameters were accepted by our IRB given the very low risk associated with the study, and the fact that any student who was not yet 18 years old had been given parental permission to live on their own, and participate in undergraduate life.

All anonymized data will be provided upon reasonable request from researchers for academic, non-commercial research.

### Participants

First-semester undergraduate students (*N* = 81) were recruited from orientation classes at a Midwest liberal arts college. There were 46 (56.8%) females and 35 (43.2%) males in the sample, which was predominantly Caucasian (93.8%). Participants’ mean age was 18.26 (*SD* = .49) years. To participate in the study, students used either their own iPhone (*n* = 33; 40.7%) or iPod Touch (*n* = 47; 58.0%); owning an iPhone or iPod Touch was a criterion for eligibility. Most users (75.9%) had owned their device for at least 6 months (*M* = 1.44 yrs; *SD* = 1.07), and, on average, participants reported using their device 87 min (SD = 103) per day. Previous research on this campus had revealed that approximately 40% of incoming freshmen owned iPhone or iPod Touch devices.

### Instruments

#### Demographic questionnaire

We used a questionnaire to gather information related to participants’ gender, age, race, and the frequency and duration of their iPhone/iTouch usage.

#### iHabit™ application

**Figure 1:**
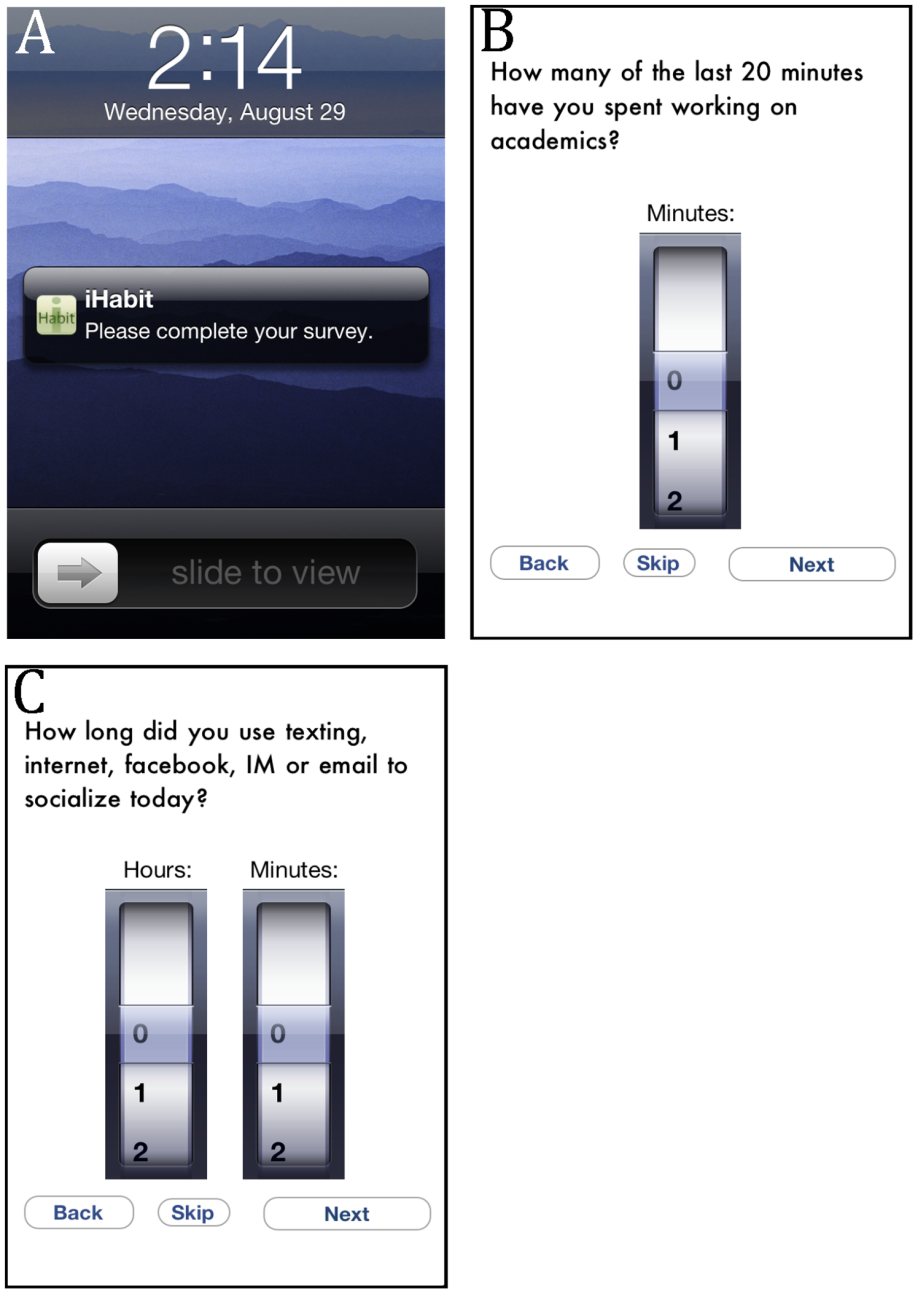
Screen captures of (A) iHabit’s visual check-in notification, (B) a representative check-in question, and (C) a representative end-of-day question.

iHabit™ is an iOS application (“app”) that runs on iPhones, iPod Touches and iPads. It was designed as an EMA app that could be downloaded by participants for free from iTunes (https://itunes.apple.com/us/app/ihabitsurvey/id440108724?mt=8), and would allow researchers to easily conduct EMA studies (www.emaresearch.org). The app platform allows alerts to be randomized during researcher-specified hours of the day. Users responding to visual notification and/or an alarm are presented with a series of “check-in” questions designed by the researcher, and provide data using their devices’ touchscreen (see Figure 1A). Response options include text entry, Likert type responses, multiple choice options, number wheels, and slide bars. Respondents can also respond to “end-of-day” questions, much like an electronic daily diary, e.g., [33]. Unlike check-in questions, end-of-day questions are initiated by the user and capture data that is more general in nature (e.g., how the day went). Finally, a “free response” feature allows respondents to submit information they believe is relevant to the study but may be missed by static questions. All data is time-stamped, automatically stored on users’ devices, and uploaded to a server when a WiFi signal is available, which could potentially occur after data collection is complete. Data can be registered and stored on the device at any time regardless of whether a user has a data plan or a WiFi signal at the time of data collection. The only time a WiFi connection is required is when the app and question packet are uploaded, and when the data is needed at the end of the study. Data is uploaded to a secure server that the researcher can access through the web using a personalized pin and password.

For the present study, iHabit was programmed to notify users 5–7 times per day (*M* = 6.76), at randomized times during specific hours of the day between 6 a.m. and 11 p.m. Respondents were asked check-in questions that focused on what the user had been doing in the last 20 minutes. For example, participants were asked, “How many of the last 20 minutes have you spent working on academics?” (see Figure 1B). Users responded using a number wheel with values ranging from 0 to 20 minutes. In addition to academics, students were asked the same kind of question about their time spent playing sports/exercising, socializing in person, recreationally using electronics, and wasting time. With respect to the last question, we purposefully left what should be considered “wasted time” up to participants’ perceptions. During orientation, we did, however, indicate that wasted time should be generally thought of as time spent doing something other than what they felt they should be doing.

The app was also programmed with end-of-day questions that assessed respondents’ use of time throughout the day. At the end of the day we asked: “How long did you study today (outside class time)?”; “How long did you use texting, internet, facebook, IM, or email to socialize today?”; “How long did you exercise today?”; and “What percentage of your time was well spent?” (see Figure 1C). Responses were entered in hours and minutes using two number wheels.

It should be noted that, given the current capabilities of the platform, once a question packet is programmed it cannot be altered while the study is in process. The only way to make modifications once a study is in process is to program a new question packet, and have participants download and use the new packet.

#### Posttest questionnaire

A posttest questionnaire was developed to assess students’ perceptions of how they spent their time during the semester, and their perceptions of how using the app had affected them. Participants were asked to estimate the average percentage of time they spent each day on the five different activities that had been assessed by EMA: (i) academics, (ii) playing sports/exercising, (iii) socializing in person, (iv) recreationally using electronics, and (v) wasting time. Participants were also asked to rate the extent to which “The iHabit app made me more aware of how I spend my time” and “I changed how I spent my time in response to the app.” Seven point Likert-type response options were provided for these two questions with anchors of “strongly disagree” and “strongly agree”.

### Procedure

The experiment was one of several learning opportunities offered to students in an orientation course. Students who owned a usable device and expressed interest in the study were told that their participation would be one option among several that would count toward credit in the course. Those in the experimental group were, additionally, given a $5 gift card after week 8 to incentivize them to (a) bring in their devices so we could verify that they were receiving auditory alerts (a few older generation iPhones required a setting change to allow for auditory alerts, which detracted from response rates), and (b) continue responding. No other incentives were given to participants.

All participants attended one of five orientation sessions in which they were informed about what participation would entail, completed a demographics questionnaire, and were assigned to the experimental (app) or control (no app) group. A random number generator was used to provide a random number to each participant. Approximately 60% of the random numbers were selected for the experimental group in an effort to increase the number of participants providing EMA data.

Participants assigned to the control group were dismissed after completing the demographics measure. Those in the experimental group remained in order to download the app to their device. After practicing one set of check-in questions, they were instructed to use the app for the coming week (week 3 of the semester), and again during weeks 8 and 14, by responding to check-in questions whenever they received notification, as long as it was safe, and did not disrupt class. Participants were, also, instructed to complete end-of-day questions at bedtime over the three testing weeks. While, at a later time, it would have been possible for all participants to download the iHabit app from the App Store, a code–given only to the experimental group–was required for downloading the question packet for this particular study thereby preventing the control group from accessing the packet. As an additional safeguard, device identification numbers, which are automatically encoded with each data point, were collected from participants to ensure that all collected data was generated from the experimental group and that the control group was not using the app.

At the end of the semester, participants in both the experimental group (*n* = 41; 93.2% of initial group) and control group (*n* = 20; 54.1% of initial group) completed posttest questionnaires in a classroom setting. With participant consent, we also obtained participants’ high school GPA, first semester college GPA, and college entrance exam scores (ACT or SAT scores that had been converted to ACT-equivalent scores) from the institution.

## Results

The EMA/EMI app group (*n* = 44) was comparable to the control group (*n* = 37) in mean age, family income, high school GPA, ACT scores, and length of time members had owned their devices (See Table 1). We observed no differences in the app vs. control group in terms of gender (66.76% vs. 66.82% female, respectively), ethnic makeup (97.62% vs. 94.59% White/Non-Hispanic, respectively), or employment status (29.55% vs. 32.43% employed, respectively).

**Table 1:** Experimental (app) and Control Group Demographics.

Variable	Experimental/App		Control		*t*	*p*
	*Mean*	*SD*	*Mean*	*SD*		
Age	18.25	.49	18.27	.51	.18	.86
Family Income (U.S. $)^1^	132,882	195,413	121,956	166,301	.22	.83
High School GPA	3.64	.39	3.70	.35	.84	.40
ACT Scores^2^	24.73	3.88	24.16	4.17	.63	.53
Owned device (months)	17.80	13.41	16.51	12.23	.44	.66

Notes: ^1^The mean family incomes for both groups were skewed by high income outliers. The median family income for the experimental/app group was $72,500 and the median income for the control group was $80,000.

2 For those reporting SAT scores, a formula was used to derive equivalent ACT scores.

Over the three weeks sampled the 44 participants in the app group registered 6,301 check-in question responses, and 1,072 end-of-day responses. On average, participants provided 18.84 (*SD* = 12.03; 40.1%) check-in responses in week 3; 16.06 (*SD* = 13.06; 34.2%) in week 8; and 9.15 (*SD* = 7.18; 19.1%) responses in week 14. Participants registered end-of-day responses an average of 4.16 (*SD* = 1.76; 59.4%) times in week 3; 3.65 (*SD* = 2.01; 52.1%) times in week 8; and 2.06 (*SD* = 1.18; 29.4%) times in week 14. Of our 44 participants in the experimental group, 40 (90.9%) registered responses during week 3; 27 (61.4%) registered responses during week 8; and 24 (54.5%) registered responses during week 14.

### App Users’ Estimates

**Figure 2:**
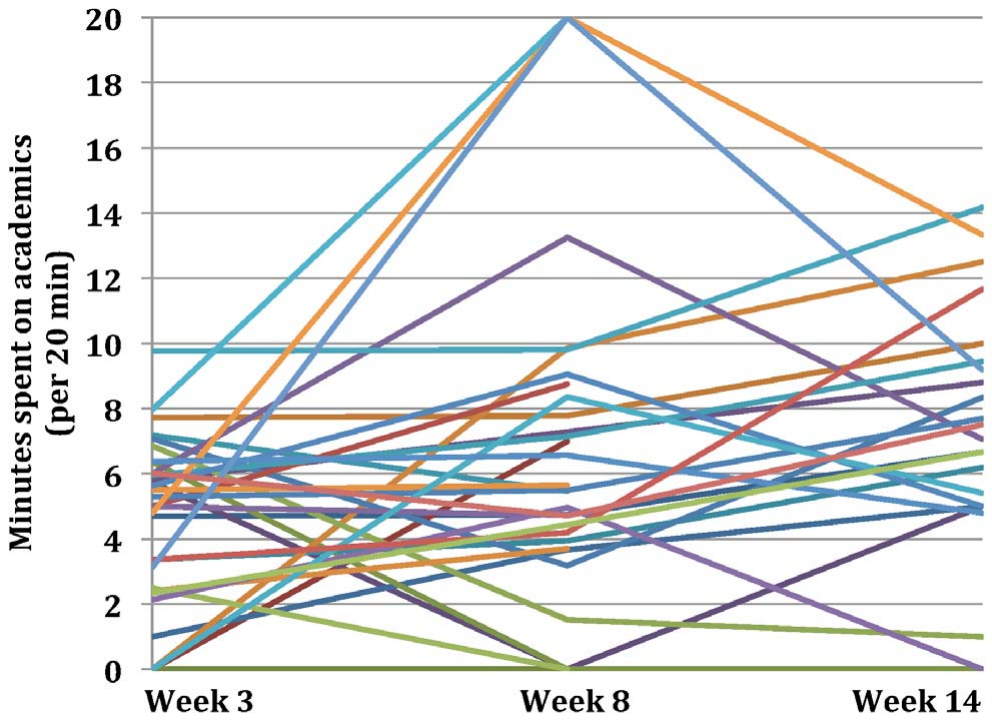
Intra-subject change in estimated time spent on academics at check-in across test weeks. Average estimated time spent on academics over the 20 minutes prior to check-in was significantly greater in weeks 8 and 14 than in week 3 (*p* = 0.05). Each participant’s average estimate during each week is represented by a colored line.

Table 2 presents the means and standard deviations for participants’ estimates of the amount of time they spent (i) socializing in person, (ii) using electronics recreationally, (iii) on academics, (iv) exercising, as well as (v) the amount of time they wasted, over the 20 minutes immediately prior to being cued to provide *check-in* responses over each of the three testing weeks (weeks 3, 8 and 14 of the semester). Average estimated time spent on academics during the later two test weeks was significantly greater than during the first test week (*F_2,21_* = 3.46, *p* = .05) (see Figure 2). Additional within-subjects ANOVAs revealed no significant change in average estimated time spent on any other activity across the three test weeks (*p’*s>.05).

**Table 2:** Participants’ estimates for how they spent their last 20 minutes at check-in points during the day.

Check-in	Week 3		Week 8		Week 14	
	Average	Ave SD	Average	Ave SD	Average	Ave SD
Socializing	42.80±14.60%	43.10±10.35%	36.70±23.50%	41.70±8.05%	39.50±24.55%	38.30±14.45%
Electronics	31.45±12.80%	36.40±9.70%	33.80±25.90%	32.27±14.10%	27.35±18.65%	35.15±15.15%
Academics	25.85±20.75%	36.00±13.90%	33.65±26.65%	39.05±12.95%	32.95±20.35%	42.8±14.95%
Waste	20.65±18.55%	25.85±12.45%	24.95±28.25%	25.70±16.80%	15.15±14.45%	24.95±19.85%
Exercise	3.85±5.35%	11.50±13.35%	5.70±13.25%	11.90±12.1%	8.15±20.35%	9.05±12.35%

Per week group means for percent of last 20 minutes spent on each activity (Average), and average within-subject standard deviation (Ave SD) for each assessment week.

When comparing estimations of time spent on the various activities within a test week, a within-subjects ANOVA revealed that average estimated time spent exercising was less than that for all other activities during weeks 3 (*F_4,43_* = 38.94, p<.01) and 8 (*F_4,31_* = 8.43, p<.01). During week 14, average estimated time spent exercising was less than that for all other activities except wasting time (*F_4,25_* = 10.89, p<.01). Additionally, during week 3, the average estimated time spent socializing was greater than that for all other activities. During week 8, the average estimated time spent socializing was only significantly greater than that for exercising; but, during week 14, average estimated time spent socializing was significantly greater than that for both exercising and wasting time.

Repeated check-in assessments of how participants spent their time allowed us to calculate *within-subject* (i.e., each individuals’) *standard deviation* for time spent on a particular activity during a testing week. We compiled these to determine, on average, how much *variation* was evident in how users spent their time each week (i.e., average within-subject standard deviation). We report these for each testing week in Table 2.

Compiling within-subject standard deviations across test weeks allowed us to test whether there was a change in the average variation for time spent on a particular activity across test weeks. It also allowed us to test whether there was a higher average variation for time spent on one activity compared to another within a given test week. Within-subjects ANOVAs revealed no significant change in average within-subject standard deviation on any activity across the three test weeks (*p’*s>.05). However, within-subjects ANOVAs revealed that the average within-subject standard deviation in estimates of time spent exercising was significantly less than that for each of the other variables during week 3 (*F_4,38_* = 42.54, p<.01), week 8 (*F_4,26_* = 17.708, p<.01), and week 14 (*F_4,23_* = 38.37, p<.01). Additionally, the average within-subject standard deviation in estimates of time wasted was significantly less than that for time spent socializing, using electronics recreationally and on academics during week 3. During week 8, the average within-subject standard deviation in estimates of time wasted was significantly less than that for socializing and academics, while during week 14 the average standard deviation in estimates of time wasted was only significantly less than that for academics.

Table 3 presents the means and standard deviations for participant *end-of-day* estimates for time spent (i) socializing, (ii) studying, and (iii) exercising, as well as (iv) percent of time well spent during the three testing weeks. There was no significant difference in any of the average end-of-day estimates across the three test weeks (*p’*s>.05). As with check-in questions, we were able to calculate participants’ average within-subject standard deviation for end-of-day estimates across the three testing weeks. These are also reported in Table 3. Within-subjects ANOVAs revealed no significant change in average within-subject standard deviation on any of the end-of-day estimates across the three test weeks (*p’*s>.05).

**Table 3:** Participants’ estimates for hours of time spent each day.

End-of-day	Week 3		Week 8		Week 14	
	Average	Ave SD	Average	Ave SD	Average	Ave SD
Social Media	2.2±1.22 hr	.59±.50 hr	1.91±1.17 hr	.53±.28 hr	1.64±1.17 hr	.66±.42 hr
Studying	1.65±1.22 hr	1.16±.85 hr	1.67±1.17 hr	1.17±.98 hr	2.29±1.84 hr	1.21±1.01 hr
Exercise	.47±.66 hr	.31±.22 hr	.30±.46 hr	.25±.32 hr	.53±.59 hr	.32±.31 hr
Well-spent	66.28±16.75%	11.97±6.19%	65.94±16.05%	10.54±4.55%	70.49±20.15%	9.24±12.33%

Group means (Average) and average within-subject standard deviation (Ave SD) for each assessment week.

### Posttest Estimations of Time Spent, & Correlations with App Responses & GPA

**Figure 3:**
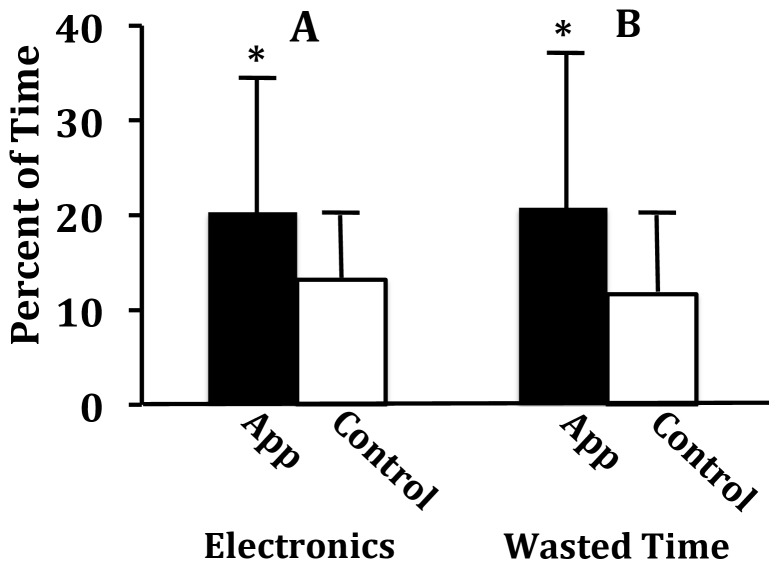
Means and standard deviations for posttest estimations of percentage of time (A) spent recreationally using electronics and (B) wasted throughout the semester. The app group estimated spending more time using electronics recreationally and wasting more time than controls (*p*<.05; statistical significance denoted by an asterisk).

A posttest questionnaire revealed 80.49% of app users mildly to very strongly agreed that using the app made them more aware of how they spent their time (*M* = 5.37; *SD* = 1.43, on a 7-point Likert scale). There was a weak positive correlation between self-reported change in awareness and number of check-in prompts responded to across the three test weeks (*r*_40_ = .33, *p* = .02). Additionally, while there was no correlation between participants’ ratings for how much the app changed their self-awareness and end-of-day estimates of time spent using electronics to socialize in week 3 (*r*_31_ = .01, *p*>.05), there was a negative and increasingly stronger correlation between the two variables in week 8 (*r*_19_ = −.46, *p* = .02) and week 14 (*r*_13_ = −.68, *p* = .003). Further, app users estimated spending significantly more time using electronics recreationally (*M* = 20.34%, *SD* = 16.44%) than did controls (*M* = 13.20%, *SD* = 7.16; *t_53_* = 2.36, *p* = .02, *d* = .65). They also estimated wasting more time (*M* = 20.78%, *SD* = 17.71%) than did controls (*M* = 11.66%, *SD* = 8.67%; *t_57_* = 2.66, *p* = .01, *d* = .70) (see Figure 3). Interestingly, end-of-semester GPA had a significant negative correlation with estimated time spent using electronics recreationally (*r_39_* = −.35, *p* = .01) and time wasted (*r_38_* = −.49, *p* = .001) among app users, but not among controls (*r_18_* = .06, *p* = .40, *r_17_* = .15, *p* = .27, respectively). No differences were found between the app users and controls in estimated time spent on academics, socializing in person, or exercising (*p*’s>.05).

**Figure 4:**
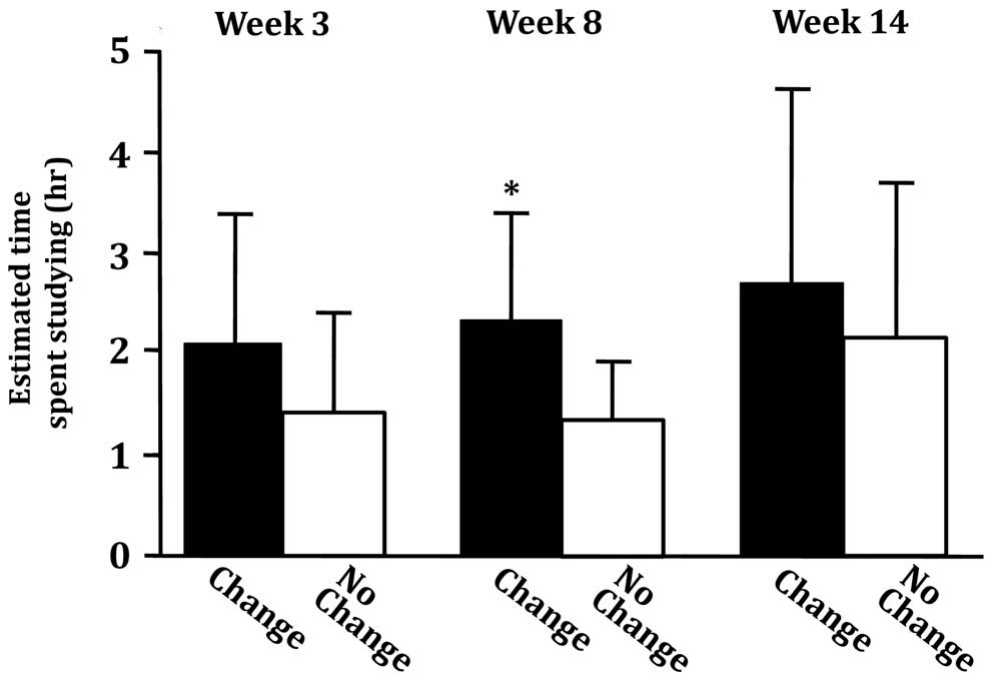
Means and standard deviations for end-of-day estimates of time spent studying during the three testing weeks for participants who reported changing their behavior as a result of app use (“Change”) and who reported no change (“No Change”) in the posttest questionnaire. Average end-of-day estimates of time spent studying over week 8–but not weeks 3 or 14 (*p*’s>,05)–was significantly higher for the group that reported changing their behavior as a result of using the app than for the group that reported no change (*p*<.01; statistical significance denoted by an asterisk).

In addition to indicating that app use made them more aware of how they spent their time, 43.90% of participants mildly to very strongly agreed that they changed their behavior as a result of app use (*M* = 3.83; *SD* = 1.72, on a 7-point Likert scale). During testing, two participants provided unprompted “free responses” indicating that app use was making them consider changing their behavior. (All participants who indicated that they had changed their behavior as a result of app use also indicated that app use had made them more aware of how they spend their time, and the correlation between reported levels of awareness and behavior change was significant, *r*_39_ = .67, *p*<.001). Consistent with these self-reports, while no difference was observed between the two groups during week 3 (*p*>.05), the group that reported changing as a result of app use had a significantly higher average end-of-day estimate of time spent studying over week 8 (*M* = 2.22 hr, *SD* = 1.26 hr) than the group that reported no change (*M* = 1.18 hr, *SD* = .66 hr; *t_20_* = 2.34, *p* = .03, *d* = 1.05) (see Figure 4). By week 14 (the week before finals) the average estimated time spent studying had risen amongst the “no change” group to a level comparable with the “change” group (no change: *M* = 2.14, *SD* = 1.60, change: *M* = 2.64, *SD* = 1.98, *p*>.05). Further, there was a significant positive correlation between individuals’ reports of change and estimated time spent studying during the second week of app use (*r_20_* = .45, *p* = .02), but not during the first (*r_32_* = .23, *p* = .10) or third (*r_13_* = .18, *p* = .26). Finally, indicating that one had changed one’s behavior as a result of app use was negatively correlated with posttest estimates of wasted time (*r_39_* = -.35, *p* = .01).

## Discussion

Using the EMA/EMI app enabled us to sample how undergraduates used their time by randomly asking them how many of the last 20 minutes they spent engaging in various activities during certain hours of their daily lives. It, thus, allowed us to ask students about their activities in real-time; thereby gathering data concerning details they would not remember long-term (e.g., amount of time spent doing *x* during a 20 min period), and minimizing recall bias. It, also, enabled us to measure intra-subject variability over a week–as well as changes in variability between sample weeks throughout the semester–in an ecologically valid way that was easily, and inexpensively, incorporated into the lives of participants. It, thus, enabled us to examine intra-subject changes as the semester progressed; and as participants had more experience with being randomly queried about how they were, in that moment, spending their time. As observed, not unexpectedly, according to their estimates, most students increased the amount they were studying as the semester progressed (see Figure 2). We were, further, able to examine the data collected using the app as it pertained to posttest questionnaire responses and semester GPA, which yielded findings indicating that using the app can promote self-awareness (see Figure 3).

The app allowed us to compare participant estimates for how they spent their time, as well as average within-subject standard deviations, both within and across testing weeks. As mentioned above, we observed that, as the semester progressed, participants estimated spending more time on academics when responding to check-in prompts during the day. An increase was not observed when, at the end of the day, participants were asked to estimate hours spent studying throughout the day. Additionally, the app allowed us to see that there was more variability in estimates for time spent engaging in certain activities than others during a given testing week (e.g., less variability in time wasted than that for time spent socializing during week 8). Further, we were able to see that some of these differences did not persist from one testing week to the next (e.g., differences in variability in estimated time wasted and spent socializing). While none of these findings were necessarily unexpected, or particularly surprising, they illustrate how the app can be used to (a) examine changes in a variable across test weeks as well as (b) differences between variables within a test week. They, likewise, illustrate how the app can be used to (c) examine changes in the *variability* of a construct across test weeks as well as (d) differences in the *variability* between constructs within a test week. In short, our within-subject standard deviation findings illustrate the app’s utility for examining intra-subject variability, and capturing the fluctuation of a construct over time.

Considering that our participants had not expressed interest in changing how they spent their time–and were not made aware that being asked about time spent may promote changes in awareness and behavior–a relatively large percentage reported that app use promoted greater awareness of how they spent their time (80.49%); and some reported even changing how they spent their time (43.90%). In fact, during assessment, we had two participants use the “free response” feature of the app to provide an unprompted note indicating that using the app was influencing their awareness and behavior. While there are certain limitations that come with relying on self-reports (e.g., the influence of subject-expectancy effects, optimism bias, selective perception, self-enhancement), a number of our observations, taken together, indicate that the changes reported by participants’ are reflective of actual changes in self-awareness.

First, at the end of the study, the app group reported recreationally using electronics more, and wasting more time, than controls did. And, unlike controls, their reported recreational use of electronics and wasted time was related to their GPA at a strength comparable to two of the best predictors of first-year college performance: high-school GPA (*r* = .48) and ACT scores (*r* = .43) [34], [35]. Since there was no difference between the groups in high school GPA or ACT scores, this indicates that app use precipitated greater accuracy in estimating the amount of time spent using electronics recreationally, and the amount they engaged in a general form of wasted time that is antagonistic to academic performance.

Second, while there was no correlation between time spent on electronics during the first week of app use and self-reported change in awareness of time spent due to app use, there was a negative correlation between these two variables in the later weeks; after participants had used the app for at least a week. Some of the strengthening of this correlation may be an effect of attrition (especially in week 14), but it is unlikely that this could account for the magnitude of change observed. Taken in the context of our other findings, the strengthening of this correlation in the later weeks provides further evidence that app use promoted changes in awareness of time spent; and–by indicating that those who reported a change in awareness were likely to spend less time using electronics *only after having used the app*–it, also, suggests the possibility that app use may have promoted changes in how some participants spent their time.

Third, while there was no correlation between time spent studying during the first, or third, weeks of app use and self-reported behavioral changes due to app use, there was a correlation between the two variables during the second week of use. Additionally, those who reported changing their behavior as a result of app use indicated higher-levels of studying during the second week of app use, at mid-semester, but not during the first and third weeks of app use–toward the beginning and end of the semester, respectively (see Figure 4). Further, reporting that one had changed one’s behavior as a result of app use negatively correlated with reported percentage of time wasted throughout the semester, which (as already observed) was a predictor of end-of-semester GPA. Together, these observations indicate that app use prompted greater self-awareness concerning time-management; and again, though not conclusive, suggests that app use may have led some participants to make changes in how they spent their time, thus promoting positive changes in their behavioral patterns (namely, spending more time studying *earlier* in the semester).

Shiffman [20], and others [36], have noted that the impact of EMI is maximized under conditions that involve high levels of participant motivation and capacity to achieve a desired change (control). Our current study indicates that using the app promoted a higher degree of self-awareness, which other studies have indicated can promote positive change, cf. [19], [37]. Taken together, this suggests, that the app may have the potential of enhancing positive change, when used for that purpose by individuals who are motivated and capable–as many of our participants, being individuals seeking higher education, likely were. This suggestion is in keeping with a number of studies demonstrating the effectiveness of using other forms of EMI in clinical, and health-related, contexts [20], [23], [24]. Given our EMA/EMI app platform:

(1) solves many of the logistical problems which have limited the use of EMA/EMI,

and

(2) can be easily used to gather data pertaining to enumerable research questions and/or to modify self-awareness regarding enumerable thought patterns and/or behaviors,

use of this app (or others like it) is a viable way of expanding a more acute and powerful approach to psychological assessment; and it presents a way of promoting self-awareness, which has the potential of promoting positive change in various behaviors and contexts. We, along with other researchers, are, currently, exploring the use of the app in clinical, educational and religious settings for these purposes, e.g. [39].

Through this study it has, additionally, become apparent that certain limitations, or challenges, come with using the app. Several participants indicated not hearing the auditory prompt, and, as a result, missed several check-in prompts. This was a result of their device “Notification” settings not being set to allow for auditory prompts. Though this impacted response rates, once the settings were appropriately adjusted, this problem disappeared. Particular to our study, students were instructed to turn off, and not reply to, alerts during class, which, also, likely impacted response rates. Attrition posed another difficulty. By week 14, only 60% of participants who were responding in week 3 were still providing responses, and at a reduced response rate. Yet, despite attrition and relatively low response rates, the amount the experimental group did respond was enough to impact their self-awareness as indicated by their more accurate self-assessment regarding a form of wasted time (see above). Nevertheless, our level of attrition indicates that, for extended EMA/EMI use, participants will need to be sufficiently motivated by either a desire for change or sufficient compensation. Our participants had not expressed any desire to change how they spent their time, and were only compensated by a few credit points in an orientation course, which they could earn by other means, and a $5 gift card. One way to better incentivize participation in EMA/EMI studies is to set up a pay system wherein participants are paid a small amount for each response, cf. [38], or receive incentive pay if they meet an established response rate, cf. [40]. Another limitation was that the app only runs on Apple devices. As a result, our applicant pool was decreased, and we were also not able to rule out the possibility that those who purchase Apple products may respond differently to EMI than those who gravitate toward other handheld devices. To resolve this issue we are currently working on developing an app that works on Apple, Android and Window Mobile platforms.

A further limitation particular to the present study was that our participants were largely of a select demographic: American Caucasians in their late teens, with a middle-to-high social economic status, attending a Christian liberal arts college. It has yet to be seen whether other demographics will respond similarly to using the app. One possibility is that, given the Christian ethos of the academic community, and that our participants were motivated to seek an undergraduate education, our participants may have been more disposed to being impacted by using the app than certain other demographics.

In 2011, there were 93.10 million smartphones users just in the U.S., and it is estimated that this number will increase to 192.40 million by 2016 [41]. It is further estimated that 10 billion smartphones and tablets will be used worldwide by 2016 [42]. These devices are, thus, becoming ideal tools for collecting real-time psychological data, and for promoting positive change in real-time in a way that seamlessly integrates into contemporary life. However, as indicated above, toward these efforts several issues require further study. First, though there has been an initial study examining response rates for various cell phone approaches to EMA in comparison to PDA approaches [43], issues pertaining to attrition, response rates and effective incentivizing across various approaches to EMA need further examination. Second, more research needs to be done on the effectiveness of EMI, and, on the related issue, of whether EMA causes *reactivity* (i.e., biasing effects as a result of measurement). Initial work has been done on the reactivity of self-monitoring, and some studies suggest that, under certain conditions (e.g., when individuals are motivated and capable of changing), reactivity results from EMA, cf. [37]. Our current study indicates that, amongst a relatively motivated demographic (undergraduate students), EMA promotes change in self-awareness. And, in some cases, this may prompt behavioral change. But more work is needed in this area. To our knowledge, no study has examined the issue of behavioral change in response to EMA/EMI with regard to personality traits, stages of change [44], having certain motivational or affective states, and/or certain demographics. These lines of study would be of interest to anyone interested in using EMA or EMI. Now, there is an app for that.
